# Calculating the Wasserstein Metric-Based Boltzmann Entropy of a Landscape Mosaic

**DOI:** 10.3390/e22040381

**Published:** 2020-03-26

**Authors:** Hong Zhang, Zhiwei Wu, Tian Lan, Yanyu Chen, Peichao Gao

**Affiliations:** 1 Faculty of Geosciences & Environmental Engineering, Southwest Jiaotong University, Chengdu 611756, China; 2 State Key Laboratory of Earth Surface Processes and Resource Ecology, Beijing Normal University, Beijing 100875, China; 3 Faculty of Geographical Science, Beijing Normal University, Beijing 100875, China; 4 Department of Land Surveying and Geoinformatics, The Hong Kong Polytechnic University, Kowloon, Hong Kong

**Keywords:** landscape, configuration, Boltzmann entropy, Wasserstein metric, software tool, information entropy, configurational entropy, compositional entropy

## Abstract

Shannon entropy is currently the most popular method for quantifying the disorder or information of a spatial data set such as a landscape pattern and a cartographic map. However, its drawback when applied to spatial data is also well documented; it is incapable of capturing configurational disorder. In addition, it has been recently criticized to be thermodynamically irrelevant. Therefore, Boltzmann entropy was revisited, and methods have been developed for its calculation with landscape patterns. The latest method was developed based on the Wasserstein metric. This method incorporates spatial repetitiveness, leading to a Wasserstein metric-based Boltzmann entropy that is capable of capturing the configurational disorder of a landscape mosaic. However, the numerical work required to calculate this entropy is beyond what can be practically achieved through hand calculation. This study developed a new software tool for conveniently calculating the Wasserstein metric-based Boltzmann entropy. The tool provides a user-friendly human–computer interface and many functions. These functions include multi-format data file import function, calculation function, and data clear or copy function. This study outlines several essential technical implementations of the tool and reports the evaluation of the software tool and a case study. Experimental results demonstrate that the software tool is both efficient and convenient.

## 1. Introduction

Raster data is widely used in diverse domains such as cartography [1,2,3], remote sensing [4,5], computer graphics [6], geography [7,8], and landscape ecology [9,10]. The content of information contained in raster data is of use in many applications [11]. For example, the information content in raster data can be used to evaluate the performance of image fusion [12,13] and is also considered a significant reference for band selection of hyperspectral images [14,15]. As a result, quantifying and understanding the information contained in raster data such as images and landscape patterns is increasingly gaining attention [16,17].

The most common method quantifying the information in raster data is through Shannon entropy (i.e., information entropy) [18,19,20,21,22,23,24,25]. Yet, Shannon entropy is incapable of fully quantifying the information in raster data because Shannon entropy is based on the probability distribution of the components of raster data [26,27,28]. In other words, Shannon entropy is a measure of the compositional information and thus cannot capture the configurational information of raster data (i.e., how the components of raster data are arranged spatially). To the best of our knowledge, there are two solutions to this limitation of Shannon entropy. The first is to improve Shannon entropy, and many improved forms of Shannon entropy have been proposed in the last few decades, e.g., [29,30,31]; see a recent review published by this journal [32]. Although most of the improved forms of Shannon entropy are valid means of quantifying configurational information, there is no definite correlation between Shannon entropy and thermodynamics [33,34,35]. The thermodynamic relevance of entropy is emphasized because, in fields such as landscape ecology [36], urban science [37], and transport geography [38], entropy is not only used for quantifying the disorder (i.e., information) but, more importantly, for linking spatial phenomenon with the underlying thermodynamic interpretations. The fact of no definite correlation means that the applications of Shannon entropy to thermodynamic interpretations of a spatial phenomenon are limited and questionable. For example, Vranken, Baudry, Aubinet, Visser, and Bogaert [35] demonstrated that the interpretations of spatial heterogeneity achieved using Shannon entropy are actually not relevant to thermodynamics. To solve such problems, Cushman [39] suggested the second possible solution, namely to revisit Boltzmann entropy and to develop methods for calculating the Boltzmann entropy of raster data.

Derived from thermodynamics, Boltzmann entropy is naturally connected with thermodynamic interpretation and characterizes the disorder (i.e., information) of a system [40,41,42]. It is theoretically capable of quantifying the information in raster data in terms of both composition and configuration and of interpreting spatial data thermodynamically [33]. In recent years, some studies have applied the Boltzmann equation to landscape ecology and proposed several methods that enable us to calculate Boltzmann entropy of landscape mosaics and landscape gradients [39,43,44,45,46,47,48]. It is important to note that although these methods were initially developed for landscape patterns, these methods apply to raster data in general, such as remote sensing images, digital elevation models, and land cover/land use maps. This is because that in essence, landscape mosaics are qualitative raster data [49], while landscape gradients are quantitative raster data [50,51].

Among all these calculation methods of Boltzmann entropy, the latest one was developed by Zhao and Zhang [47] based on the Wasserstein metric. This Wasserstein metric-based Boltzmann entropy (also referred to as configurational entropy) incorporates spatial repetition and is capable of capturing the configurational information of a landscape mosaic. However, the numerical work required to calculate the entropy is more than can be practically achieved through hand calculation, so the calculation has very limited applicability to date. It is possible, however, that digital computers could be utilized to overcome this limitation. To calculate Wasserstein metric-based Boltzmann entropy conveniently, we aim to develop a software tool that can facilitate the calculation of Wasserstein metric-based Boltzmann entropy for users.

## 2. Wasserstein Metric-Based Boltzmann Entropy

Recently, Zhao and Zhang [47] proposed the Wasserstein metric-based form of Boltzmann entropy and determined the calculation method for this entropy with a landscape mosaic. This method takes repetition in space into consideration. It is possible that mosaic cells with the same attribute (i.e., mosaic cells of the same category) are adjacent to one another such that they form a continuous space. Such a continuous space would contain repetitive information, which is taken into consideration in calculating the Wasserstein metric-based Boltzmann entropy. To reflect the role of such repetitive information, Zhao and Zhang [47] extended the original definition of Boltzmann entropy to eliminate the repetitive information of a landscape mosaic, and the extended Boltzmann entropy can be written as follows:

(1) $$S=k_{B}\ln W-\overset{m}{\underset{I=1}{\sum }}\overset{n}{\underset{j=1}{\sum }}\ln r_{ij}!$$

(2) $$=\ln N!-\overset{m}{\underset{i=1}{\sum }}\ln N_{i}!-\overset{m}{\underset{i=1}{\sum }}\overset{n}{\underset{j=1}{\sum }}\ln r_{ij}!$$

where the Boltzmann constant $k_{B}$ is set as one as reasoned by Cushman [39] (i.e., “no scaling constant is needed” in dealing with spatial data), N is the number of cells in a landscape mosaic, and $N_{i}$ is the number of cells of class i. m is the number of classes of cells, and n is the number of continuous spaces of class i. $r_{ij}$ denotes the number of cells of continuous space j.

Calculating Equation (2) directly is more likely to cause a numerical overflow. To address this problem, Zhao and Zhang [47] developed a new method based on statistical theory, namely, to use the Wasserstein metric to quantify the repetitive information in a landscape mosaic. The third term of Equation (2) can be extended by the identity transformation, and the extended form can be written as follows:

(3) $$\overset{m}{\underset{i=1}{\sum }}\overset{n}{\underset{j=1}{\sum }}\ln r_{ij}!=\left(a_{1},a_{2},\cdots ,a_{N}\right)\cdot \left(\ln1,\ln2,\cdots \ln N\right)$$

where $a_{1},a_{2},\cdots ,a_{N}$ is the coefficient of the extended logarithmic (ln1,ln2,⋯lnN).

**Figure 1:**
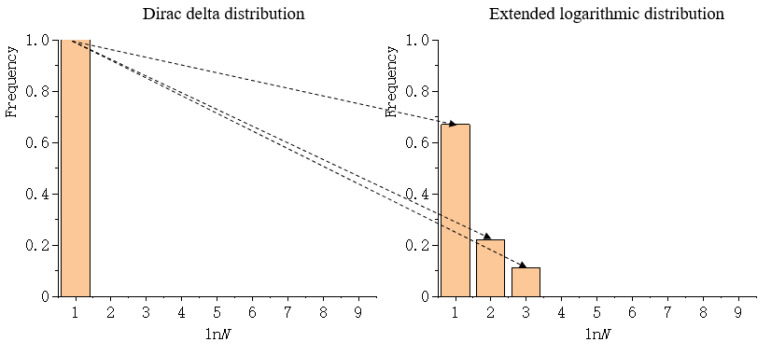
The cost of transporting mass to transform the Dirac delta distribution into the extended logarithmic distribution

The coefficient of extended logarithmic can be represented as a histogram, which is a representation of data distribution, and an estimation of the distribution of variables. Consequently, the distribution of the extended logarithmic can be represented through a histogram. However, using a histogram as a measure is inconvenient and needless. To compare the similarities of two histograms, the Wasserstein metric is of use. As Gulrajani, et al. [52] have noted, “the Wasserstein metric is informally defined as the minimum cost of transporting mass in order to transform the distribution q into the distribution p (where the cost is mass times transport distance).” Zhao and Zhang [47] regarded the Dirac delta distribution as the reference histogram. They calculated the Wasserstein metric between the distribution of the extended logarithmic and the Dirac delta distribution, as shown in Figure 1. Note that the second term of Equation (2) can also be extended by the identity transformation, and that the Wasserstein metric between the distribution of the extended logarithmic and the Dirac delta distribution can also be calculated.

The Wasserstein metrics for both class (denoted as $W_{c}$, the second term of Equation (2)) and space (denoted as $W_{c}$, the third term of Equation (2)) have been obtained. To ensure consistency with the meaning of entropy (i.e., the more uneven the distribution of the extended logarithmic, the higher the entropy), the Wasserstein metric-based relative Boltzmann entropy has been defined, and the formula can be written as follows:

(4) $$W_{dist}=\left(1-W_{c}\right)\times \left(1-W_{s}\right)$$

where $W_{c}$ is the Wasserstein metric between the distribution of the extended logarithmic of the class and the Dirac delta distribution, and $W_{s}$ is the Wasserstein metric between the distribution of the extended logarithmic of the space and the Dirac delta distribution.

The advantage of this method is that it is capable of capturing the configurational information of a landscape mosaic and avoid numerical overflow through statistical theory. The Wasserstein metric-based Boltzmann entropy considers repetitive information in continuous space to reflect different spatial configurations. Moreover, this form of entropy is based on statistical theory and thus avoids the need for a series of numerical calculation processes. This entropy has the potential to be useful in many applications, such as landscape pattern quantification [53,54] and image quality assessment [55,56]. Due to the large amount of numerical calculation required to measure this entropy, hand calculation is impractical, and accordingly, this form of entropy has very limited applicability to date. It is possible, however, that digital computers can be utilized to overcome this limitation.

## 3. Design of a Software Tool for Conveniently Calculating $W_{dist}$

**Figure 2:**
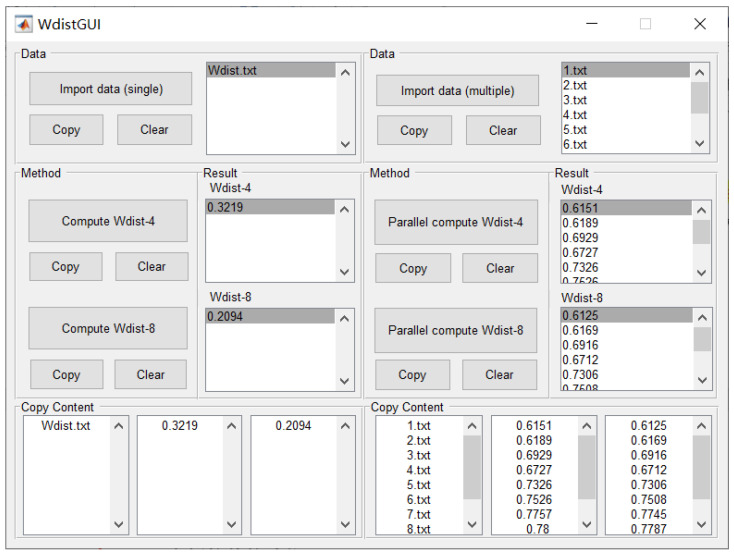
The developed software tool for conveniently calculating $W_{dist}$.

**Figure 3:**
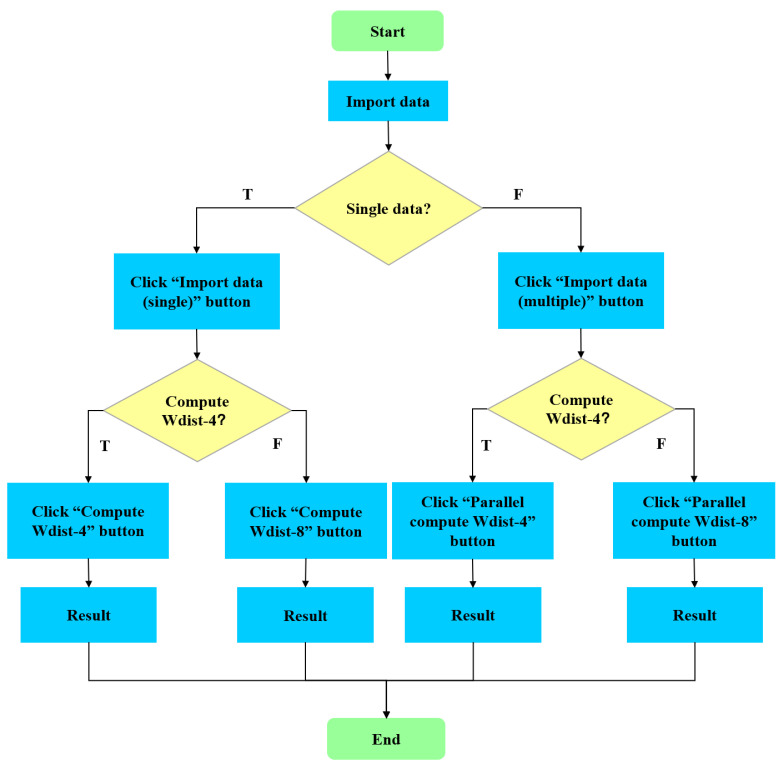
The flowchart of the developed software tool.

A challenge commonly encountered in attempts to calculate $W_{dist}$ of a landscape mosaic is the lack of a tool to calculate $W_{dist}$ easily. In order to meet this need, this study aimed to develop a software tool for conveniently calculating $W_{dist}$. The interface of the developed software tool is shown in Figure 2. The flowchart of the software tool is given in Figure 3.

**Figure 4:**
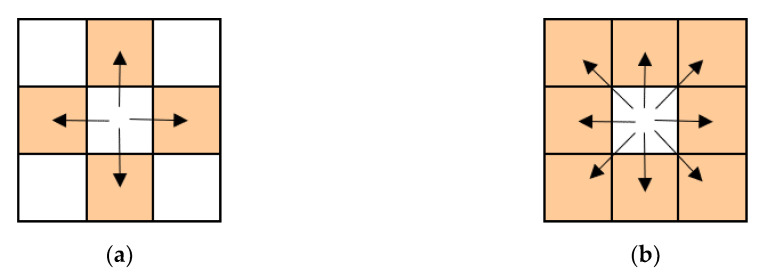
Four-neighbor connectivity (**a**) and eight-neighbor connectivity (**b**).

The functions of the software tool have been designed as follows. Its first function is to import data. As the formats of data files may vary, the software tool offers various format options for data files, including text, jpg, and bmp. Each of these formats is widely used in dealing with landscape mosaics. Additionally, as it is inefficient to import many data files one at a time, the software tool provides a function to import multiple data files simultaneously. The second function is to calculate $W_{dist}$ of the imported data. Traditionally, continuous space is determined according to four-neighbor connectivity when calculating the Wasserstein metric-based Boltzmann entropy. To enable more comprehensive statistics on continuous spaces, we made the software tool capable of determining a continuous space according to eight-neighbor connectivity (Figure 4).

**Figure 5:**
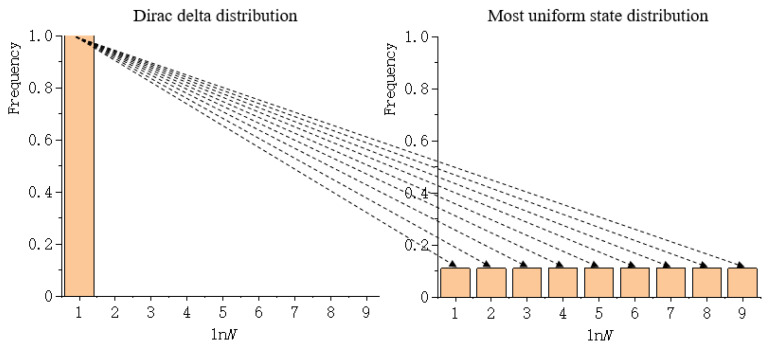
The cost of transporting mass to transform the Dirac delta distribution into the most uniform state distribution.

Comparing the Wasserstein metrics of different landscape mosaics requires calculating the normalized Wasserstein metric, i.e., dividing the Wasserstein metric between the distribution of the extended logarithmic and the Dirac delta distribution by the theoretical maximum Wasserstein metric. The theoretical maximum Wasserstein metric is the cost of transporting mass in order to transform the Dirac delta distribution into the most uniform state distribution, as shown in Figure 5. Rather than calculating $W_{dist}$ of one data file at a time (serial calculation), the software tool is capable of calculating $W_{dist}$ of multiple data files concurrently through the technique of parallel calculation. The third function is to copy or clear the imported data file name and the calculational results, and both of these are displayed in text boxes.

The design of the software tool interface follows a symmetrical layout, and the layout is divided into the left and right sides. The left side is designed for importing and calculating single data files, i.e., one at a time. The right side is designed for importing and calculating multiple data files at once. The functions of clear and copy are also symmetric on the left and right sides.

## 4. Technical Implementation of the Software Tool

The key to calculating $W_{dist}$ is calculating $W_{c}$ and $W_{s}$. In the process of calculating $W_{c}$, the software tool first counts the number of pixels (N) of each class in the given landscape mosaic through the “tabulate” function in MATLAB. Second, the program extends lnN! to the form of (ln1+ln2+⋯+lnN) and counts the number of lnN. Third, to calculate the cost of transforming the Dirac delta distribution into the distribution of the extended logarithmic, the program multiplies the transported mass (the proportion of lnN) by the transported distance (lnN−ln1). Note that, as the position of the bin of Dirac delta distribution is ln1, which is equal to 0, we abbreviate the transported distance between the two bins as lnN. Finally, the program divides the cost by the theoretical maximum cost to obtain the normalized version.

In the process of calculating $W_{s}$, the program first sets the pixel value of the first class of continuous space to be searched to one and the pixel value of the remaining classes to 0, because the program determines each class of continuous space in turn. Second, the program stores the reset pixels as the new landscape mosaic and determines the continuous space of the new landscape mosaic through the “bwlabel” function in MATLAB. Parameters of the “bwlabel” function can be set to four or eight, corresponding to four-neighbor connectivity or eight-neighbor connectivity, respectively. Third, the program counts the number of pixels (N) of continuous space in each class through the “tabulate” function in MATLAB. Then, the program extends lnN! to the form of (ln1+ln2+⋯+lnN) and counts the number of lnN. Fourth, as in the third-to-last step in calculating $W_{c}$, the program multiplies the transported mass (the proportion of lnN) by the abbreviated transported distance (lnN) to calculate the cost of transforming the Dirac delta distribution into the distribution of the extended logarithmic. Finally, the program divides the cost by the theoretical maximum cost to obtain the normalized version. After $W_{c}$ and $W_{s}$ have been calculated for the given landscape mosaic, both can be substituted into Equation (4) to get $W_{dist}$ of the landscape mosaic.

As counting the number of mosaic cells (N) of continuous space in each class can be performed independently, parallel techniques, e.g., [57,58,59,60] can be used to save time in calculating $W_{dist}$ of a single data file by using the “parfor” tool in MATLAB. Moreover, the calculation of $W_{dist}$ of each data file is independent, and its calculation for multiple data files, in turn, is a time-consuming process. To save time when calculating this figure for multiple data files, the technique of parallel calculation can be used by means of the “parfor” tool.

This software tool can be used to integrate import data function, calculation function, and output function into the interface through the user-friendly graphical user interface (GUI) tool in MATLAB. The key to using this GUI tool is to write a code that implements the function of the software tool into the callback function of control. Consequently, different control events such as click events can be used to trigger different software tool functions to achieve human–computer interaction. The transfer of variables between different attributes is available by setting the “handles” parameter in the callback function. For example, after triggering the function by which data files are imported, the file name of the data can be stored in the attribute of the “userdata” of the control, and the program can call the file name of the data through accessing the “userdata” of the control. This software tool (including manual and pseudocode) can be found in the Supplementary Materials.

## 5. Evaluation

**Figure 6:**
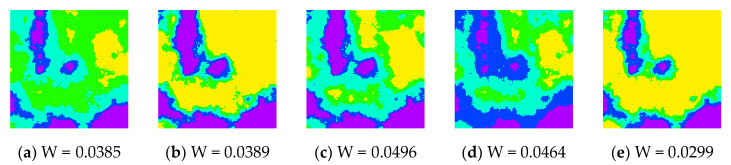
Representative examples of the 50 simulated landscape mosaics.

To evaluate the efficiency of the developed software tool in calculating the Wasserstein metric-based Boltzmann entropies of multiple data files with parallel calculation and serial calculation, we conducted a series of experiments. The first experimental dataset consisted of 50 simulated landscape mosaics built using the program Qrule [61], each of which contained 128×128 pixels of simulated data. Representative simulated landscape mosaics are shown in Figure 6.

**Figure 7:**
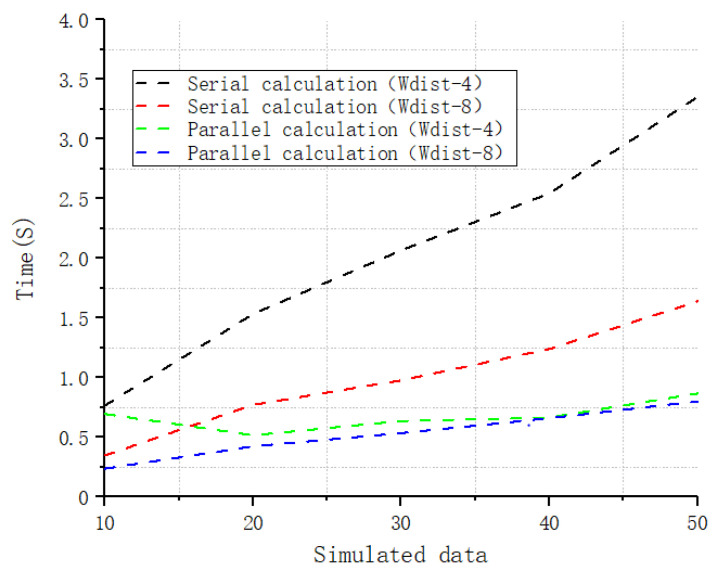
The time required to calculate the Wasserstein metric-based Boltzmann entropies of simulated datasets ranging in size from 10 to 50 landscape mosaics at 10-mosaic intervals.

The Wasserstein metric-based Boltzmann entropy was calculated using four-neighbor connectivity and eight-neighbor connectivity in an identical operating environment (Intel Core i7-8750H CPU @ 2.20 GHz, 12.00 GB RAM, and 64-bit Windows 10). Whichever connectivity is adopted, the time complexity of the calculation is O(MNC), where M×N and C are the size of and the number of categorical classes of the landscape mosaic in question, respectively. In the experiment, we calculated datasets with 10, 20, 30, 40, and 50 landscape mosaics both in the parallel and in serial mode using the developed software tool. The time required is shown in Figure 7. It can be seen from this figure that the time required by the parallel calculation mode is shorter than that required using serial calculation when there are multiple data files. The advantage of parallel calculation becomes increasingly significant along with the number of data files.

**Figure 8:**
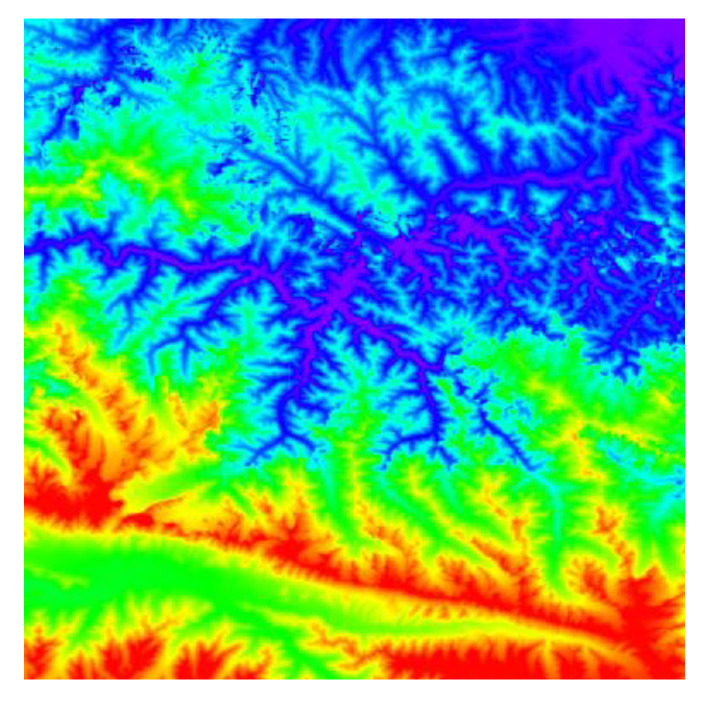
The digital elevation model (DEM) obtained from the Geospatial Data Cloud site.

A second experiment was conducted to evaluate the efficiency of the developed software tool in calculating the Wasserstein metric-based Boltzmann entropy of a single data file with parallel calculation and serial calculation. This experimental dataset consisted of a digital elevation model (DEM) obtained from the Geospatial Data Cloud site (http://www.gscloud.cn) with a size of 1024×1024 pixels, as shown in Figure 8. In the experiment, a DEM is regarded as a landscape mosaic where the categories of cells are formed according to elevations.

**Figure 9:**
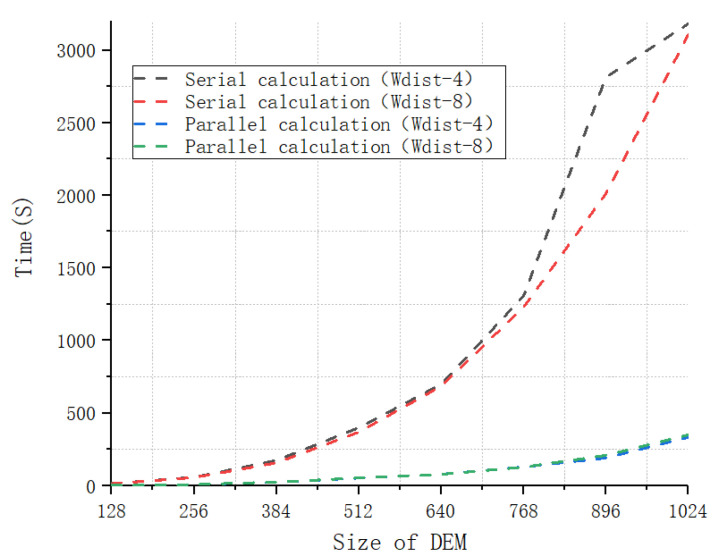
The time required by the software tool to calculate the Wasserstein metric-based Boltzmann entropies of DEMs of different sizes.

Seven additional DEMs were obtained by changing the size of the original simulated landscape mosaic. Their sizes ranged from 128×128 pixels to 1024×1024 pixels. The Wasserstein metric-based Boltzmann entropies of the eight DEMs were calculated using four-neighbor connectivity and eight-neighbor connectivity in an identical operating environment. The calculation times required to analyze these data are reported in Figure 9. It can be seen from this figure that the time grows exponentially with the size of a landscape mosaic. This fact is consistent with the analysis of time complexity. In addition, the time required by serial calculation is far higher than that required by parallel calculation. The increase in the time required for serial calculation that occurs as the size of the DEM increases is faster than the growth in the time required for parallel calculation.

## 6. Case Studies

In this section, we present two case studies to elaborate on how to use the software tool and the usability of the Wasserstein metric-based Boltzmann entropies.

**Figure 10:**
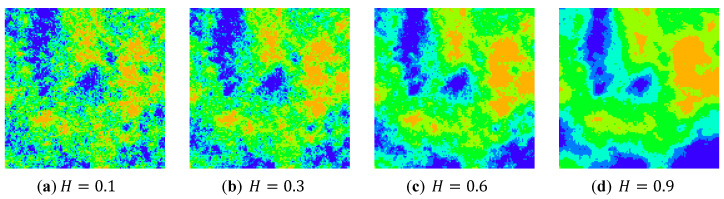
A set of simulated landscape mosaics with different Hurst exponent values (H).

In the first case study, a set of simulated landscape mosaics was built using the program Qrule [61]. All simulated landscape mosaics have the same number of classes (6 classes) and the same proportion of data in each class. Their Hurst exponent values (H), however, are different, being 0.1, 0.3, 0.6, and 0.9, respectively, as shown in Figure 10. The Hurst exponent value describes the disorder or aggregation of cells within a landscape mosaic. In other words, a lower H value indicates that the cells have a higher disorder or lower auto-correlation. Accordingly, the four simulated landscape mosaics exhibit a downward trend of the disorder. If the Wasserstein metric-based Boltzmann entropies is a measure of disorder, their values should decrease from Figure 10a–d.

Before using the software tool to calculate $W_{dist}$ of the simulated landscape mosaics in Figure 10, we saved these simulated landscape mosaics as text files. We opened the software tool and imported these text files by clicking the “Import data (multiple)” button. We were then able to calculate all $W_{dist}$ metrics of the simulated landscape mosaics using four-neighbor connectivity and eight-neighbor connectivity by clicking the “Parallel compute Wdist-4” and “Parallel compute Wdist-8” buttons, respectively. Finally, we copied these text files’ names and the calculational results into the corresponding text boxes. The calculation results are shown in Table 1.

The results indicate that the simulated landscape mosaics with different spatial structures have different $W_{dist}$. In particular, both $W_{dist-4}$ and $W_{dist-8}$ are capable of quantifying the disorder of the simulated landscape mosaics. As shown in Table 1, both $W_{dist-4}$ and $W_{dist-8}$ exhibit a downward trend from Figure 10a to Figure 9d. The greater value $W_{dist-4}$ (or $W_{dist-8}$) has, the more disorder the simulated landscape mosaic is. In addition, it can be seen that the developed software tool can be conveniently used for the calculation of both $W_{dist-4}$ and $W_{dist-8}$.

**Figure 11:**
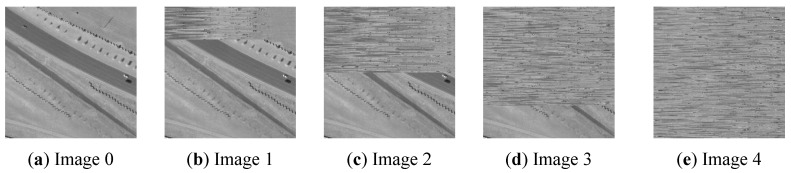
A gray-level remote sensing image (**a**) and four simulated images (**b**–**e**).

In the second case study, we applied the Wasserstein metric-based Boltzmann entropies to quantify the dissimilarity between digital images. To generate digital images of different similarities, we followed the method by Gao, Wang, Zhang, and Li [15]. First, we prepared a gray-level remote sensing image (Image 0) of size 1024×1024 pixels, based on a dataset for vehicle detection in aerial imagery [62], as shown in Figure 11a. Then, we generated four images (Images 1–4) by randomizing the first 256, 512, 768, and 1024 rows of Image 0, as shown in Figure 11b–e. Theoretically, the dissimilarity between the seed image and the other images should be increasing from Image 1 to Image 4.

Here, we propose to use the absolute difference in Wasserstein metric-based Boltzmann entropy to characterize such an increasing trend. The results are shown in Table 2. It can be seen from this table that both $\left|\Delta W_{dist-4}\right|$ and $\left|\Delta W_{dist-8}\right|$ show an upward trend from the dissimilarity between Image 0 and Image 1 to that between Image 0 and Image 4, demonstrating that the dissimilarity has been successfully characterized. These results also showed the potential of the Wasserstein metric-based Boltzmann entropies in processing remote sensing images such as band selection.

## 7. Discussion

Careful readers may notice that the performance of the Wasserstein metric-based Boltzmann entropies ($W_{dist-4}$ and $W_{dist-8}$) are dependent on $W_{c}$ and $W_{s}$, as shown in Equation (4). In this section, we have a close look at the values of $W_{c}$ and $W_{s}$.with the experimental data used in the two case studies, hoping that this effort will develop a deeper understanding of $W_{dist}$. In calculating $W_{c}$ and $W_{s}$, we also distinguished four-neighbor connectivity from eight-neighbor connectivity, leading to $W_{c-4}$, $W_{s-4}$, $W_{c-8}$, and $W_{s-8}$. The results are shown in Table 3.

As shown in Table 3, all the four values of $W_{c-4}$ are different with the four landscapes used in the first case study. In particular, these four values exhibit an upward trend from Landscapes (a) to (d). Similar patterns can also be observed with $W_{c-8}$, $W_{s-4}$, and $W_{s-8}$. These facts suggest that the decreasing disorder from Landscapes (a) to (d), measured by either $W_{dist-4}$ or $W_{dist-8}$, is caused by both the composition and the configuration of mosaic cells. By contrast, for Images 0– 5, all their ($W_{c}$)s have the same value, but their ($W_{s}$)s are different, demonstrating that their difference in disorder lies not in the composition of pixels but the configuration of pixels.

## 8. Concluding Remarks

In this study, we first review Wasserstein metric-based Boltzmann entropy and its method of calculation. This form of entropy takes repetition in space into consideration and is based on statistical theory. This entropy has the potential to be useful in many applications, such as image quality assessment, but a software tool is needed for its practical use. In order to calculate the Wasserstein metric-based Boltzmann entropy conveniently, this study developed a software tool that provides many useful functions and user-friendly interface with a symmetrical layout. The software tool is capable of calculating the entropy using either four-neighbor connectivity or eight-neighbor connectivity, and it deals with calculation takes in parallel. We have carried out two case studies; one with qualitative raster data (i.e., landscape mosaics) and the other with quantitative raster data (i.e., digital images). Experimental results show that the software tool is both efficient and convenient.

We hope that this software tool will be useful in quantifying spatial data information from a new perspective and that it will contribute to advances in the development of new forms of entropy with many applications such as image processing and landscape evaluation [63].

## Figures and Tables

**Table 1:** $W_{dist-4}$ and $W_{dist-8}$ of the four simulated landscape mosaics.

Landscape	$W_{dist-4}$	$W_{dist-8}$
a	0.1611	0.1294
b	0.1345	0.1006
c	0.0829	0.0688
d	0.0601	0.0590

**Table 2:** The image dissimilarity characterized using $W_{dist-4}$ and $W_{dist-8}$.

Dissimilarity	$\left|\Delta W_{dist-4}\right|$	$\left|\Delta W_{dist-8}\right|$
Images 0 and 1	$1.2\times 10^{-3}$	$2.2\times 10^{-3}$
Images 0 and 2	$2.6\times 10^{-3}$	$5.0\times 10^{-3}$
Images 0 and 3	$3.6\times 10^{-3}$	$6.8\times 10^{-3}$
Images 0 and 4	$4.3\times 10^{-3}$	$8.4\times 10^{-3}$

**Table 3:** The details in calculating the entropies of experimental data of the two case studies.

Data	$W_{c-4}$	$W_{c-8}$	$W_{s-4}$	$W_{s-8}$
Landscape a	0.8054	0.8054	0.1720	0.3352
Landscape b	0.8055	0.8055	0.3086	0.4827
Landscape c	0.8057	0.8057	0.5735	0.6461
Landscape d	0.8058	0.8058	0.6905	0.6960
Image 0	0.6633	0.6633	0.0256	0.0396
Image 1	0.6633	0.6633	0.0221	0.0330
Image 2	0.6633	0.6633	0.0178	0.0246
Image 3	0.6633	0.6633	0.0150	0.0192
Image 4	0.6633	0.6633	0.0128	0.0146

## Supplementary Materials

The following are available online at https://www.mdpi.com/1099-4300/22/4/381/s1.
