# Supplementary material for: Calculating the Wasserstein Metric-Based Boltzmann Entropy of a Landscape Mosaic
Source: Entropy (Basel). 2020 Mar 26;22(4):381. doi: 10.3390/e22040381 (PMC7516855; doi:10.3390/e22040381)
Supplement: Supplementary file 1 [file entropy-22-00381-s001.zip › entropy-728660-supplementary/Supplementary materials/Pseudocode/Compute Wdist-4 button.docx]

Compute *Wdist-4* for the selected file

*path = handles.pushbutton1.file_path*

*data =* Read the data of *path*

*num_rows =* Compute the number of rows of *data*

*num_columns =* Compute the number of columns of *data*

*num_pixels = num_rows* times *num_columns*

**If (**both *num_rows* and *num_columns* are greater than hundred**)**

Prompt user program is calculating, please wait

**End**

*num_class_data* = Count the number of class in *data*

*tabulate_data* (1, *i) =* Count the number of pixels of the *i*^th^ class in *data*

**Parallel.For** *i* = 1 to *num_class_data*

*ln_data +=* [(ln1), (ln2), ……, ln (*tabulate_data* (1, *i*))]

**End**

*num_class_ln_data* = Count the number of class in *ln_data*

*tabulate_ ln_data* (1, *j*) *=* Count the number of the (ln*j*) in *ln_data*

*fre_ tabulate_ ln_data* (1, *j*) *=* Compute the frequency of the (ln*j*) in *tabulate_ ln_data*

**Parallel.For** *j* = 1 to *num_class_ln_data*

*w_class +=* (ln*j*) times the frequency of the (ln*j*)

**End**

**Parallel.For** *i* = 1 to *num_class_data*

*derive_data =* Set the value of the *i*^th^ class in *data* to 1 and those of other classes in *data* to 0

Search continuous space with a value of 1 in *derive_data* according to four-neighbor connectivity

**For** *p* = 1 to the number of continuous spaces with a value of 1

*tabulate_ derive_data* (1, *p) =* Count the number of pixels of the *p*^th^ continuous space in *derive_data*

*ln_ derive_data +=* [(ln1), (ln2), ……, ln (*tabulate_ derive_data* (1, *p*)]

**End**

**End**

*num_class_ln_ derive_data* = Count the number of class in *ln_ derive_data*

*tabulate_ ln_ derive_data* (1, *q*) *=* Count the number of the (ln*q*) in *ln_ derive_data*

*fre_ tabulate_ ln_data* (1, *q*) *=* Compute the frequency of the (ln*q*) in *tabulate_ ln_ derive_data*

**Parallel.For** *h* = 1 to *num_class_ln_ derive_data*

*w_space +=* (ln*h*) times the frequency of the (ln*h*)

**End**

**Parallel.For** *k* = 1 to *num_pixels*

*w_normal +=* (ln*k*) times (1/ *num_pixels*)

**End**

*Wdist-4 =* (1- *w_class*/ *w_normal*) times (1- *w_space*/ *w_normal*)

*handles.listbox2 = Wdist-4*
