# Supplementary material for: Calculating the Wasserstein Metric-Based Boltzmann Entropy of a Landscape Mosaic
Source: Entropy (Basel). 2020 Mar 26;22(4):381. doi: 10.3390/e22040381 (PMC7516855; doi:10.3390/e22040381)
Supplement: Supplementary file 1 [file entropy-22-00381-s001.zip › entropy-728660-supplementary/Supplementary materials/Pseudocode/Import data (multiple) button.docx]

Import multiple data files

*file_names* = [The name of the first selected file, the name of the second selected file,……]

*file_paths* = [The path of the first selected file, the path of the second selected file,……]

**If** the length of the *file_names* equals zero

Prompt the user to select multiple files

**else**

*handles.listbox6 = file_names*

*handles.pushbutton2 =* [*file_paths, file_names*]

**End**
