# Supplementary material for: Calculating the Wasserstein Metric-Based Boltzmann Entropy of a Landscape Mosaic
Source: Entropy (Basel). 2020 Mar 26;22(4):381. doi: 10.3390/e22040381 (PMC7516855; doi:10.3390/e22040381)
Supplement: Supplementary file 1 [file entropy-22-00381-s001.zip › entropy-728660-supplementary/Supplementary materials/Pseudocode/Import data (single) button.docx]

Import a single data file

*file_name* = The name of the selected file

*file_path* = The path of the selected file

**If** the length of the *file_name* equals zero

Prompt the user to select a file

**else**

*handles.listboxl = file_name*

*handles.pushbutton1 =* [*file_path file_name*]

**End**
